# HGFL supports mammary tumorigenesis by enhancing tumor cell intrinsic survival and influencing macrophage and T-cell responses

**DOI:** 10.18632/oncotarget.3641

**Published:** 2015-04-17

**Authors:** Nancy M. Benight, Purnima K. Wagh, Glendon M. Zinser, Belinda E. Peace, William D. Stuart, Juozas Vasiliauskas, Peterson Pathrose, Sandra L. Starnes, Susan E. Waltz

**Affiliations:** ^1^ Department of Cancer Biology, University of Cincinnati College of Medicine, Cincinnati, OH 45267, USA; ^2^ Department of Surgery, University of Cincinnati College of Medicine, Cincinnati, OH 45267, USA; ^3^ Research Service, Cincinnati Veterans Affairs Medical Center, Cincinnati, OH 45267, USA; ^4^ Susan G. Komen, Scientific Grants Manager, Dallas, TX 75244, USA; ^5^ Senior Medical Writer, Optum, Eden Prairie, MN 55344, USA

**Keywords:** receptor tyrosine kinase, Ron receptor, hepatocyte growth factor-like protein, breast cancer

## Abstract

The Ron receptor is overexpressed in human breast cancers and is associated with heightened metastasis and poor survival. Ron overexpression in the mammary epithelium of mice is sufficient to induce aggressive mammary tumors with a high degree of metastasis. Despite the well-documented role of Ron in breast cancer, few studies have examined the necessity of the endogenous Ron ligand, hepatocyte growth factor-like protein (HGFL) in mammary tumorigenesis. Herein, mammary tumor growth and metastasis were examined in mice overexpressing Ron in the mammary epithelium with or without HGFL. HGFL ablation decreased oncogenic Ron activation and delayed mammary tumor initiation. HGFL was important for tumor cell proliferation and survival. HGFL loss resulted in increased numbers of macrophages and T-cells within the tumor. T-cell proliferation and cytotoxicity dramatically increased in HGFL deficient mice. Biochemical analysis of HGFL proficient tumors showed increased local HGFL production, with HGFL loss decreasing β-catenin expression and NF-κB activation. Re-expression of HGFL in HGFL deficient tumor cells stimulated cell migration and invasion with coordinate activation of NF-κB and reduced apoptosis. Together, these results demonstrate critical *in vivo* functions for HGFL in promoting breast tumorigenesis and suggest that targeting HGFL may inhibit tumor growth and reactivate anti-tumor immune responses.

## INTRODUCTION

Breast cancer is the most commonly diagnosed cancer amongst women in the US, with approximately 12% of women expected to develop invasive breast cancer during their lifetime [1]. While treatment advances and earlier detection have contributed to a decline in death rates for breast cancer patients, 20 to 30% of patients initially diagnosed with early stage disease will develop metastatic breast cancer, many in spite of successful treatment of a primary tumor, accounting for approximately 40,000 deaths annually [1]. Thus, understanding the mechanisms that contribute to aggressive breast cancer and defining new treatment modalities that have the ability to combat the growth and spread of this disease are needed.

The Ron receptor tyrosine kinase is a member of c-Met family of receptors and is upregulated in many cancers, including breast, prostate and lung [2]. Ron expression is minimally detectable in normal epithelial cells of the mammary gland but is overexpressed in a majority of human breast cancers [2–4]. Importantly, overexpression of Ron in patient samples correlates with poor prognosis and increased metastasis in breast, ovarian and colorectal cancers [5–7]. Similar to human breast cancers, the feline form of Ron is overexpressed in about 40% of sporadic feline mammary carcinomas, documenting a similar role for Ron overexpression in breast cancers from multiple species [8]. The ligand for Ron is hepatocyte growth factor-like protein (HGFL) [9]. HGFL binding to Ron elicits tumorigenic potential by regulating epithelial cell proliferation, motility, adhesion and anoikis [10–13]. Studies from our laboratory were the first to demonstrate a functional significance for Ron in breast cancer, with loss of Ron signaling delaying tumor initiation, growth and metastasis [14]. To determine if Ron overexpression could be causative in disease progression, mice that overexpress Ron selectively in the mammary epithelium (MMTV-Ron mice) were generated [4]. In these animals, Ron overexpression was sufficient to induce aggressive breast tumors that were highly metastatic. All female mice developed breast tumors, with the majority exhibiting lung and liver metastases. Further studies have shown that Ron elicits its tumorigenic potential *in vivo* through the activation of downstream signaling pathways, including β-catenin, PI3K/Akt, MAPK, STAT3 and NF-κB [14–18]. Ron's activation of β-catenin was shown to be important for breast tumor onset, growth and metastasis, with activation of NF-κB critical for regulating tumor cell survival and angiogenesis [4, 17–19]. Thus, mounting evidence indicates that Ron overexpression is a causative factor contributing to aggressive breast cancer and metastatic disease.

Ron expression is also found on tissue resident macrophages [16, 20–25] and its activity has been associated with resolving the inflammation and supporting tissue healing after activation of the innate immune system [24, 26–29]. Recent studies have examined the importance of Ron in the polarization of tumor-associated macrophages (TAMs), where Ron signaling loss initiates a switch from a pro-tumorigenic (M2) polarization state to a classical or inflammatory (M1) state [16, 30], leading to a decrease in tumor burden. Decreased tumor growth was associated with enhanced numbers of CD8+ cytotoxic T-cells within the tumor microenvironment. Further, antibody depletion of CD8+ T-cells was able to restore aggressive tumorigenesis [16], indicating that Ron signaling in tumor-associated macrophages influences CD8+ cytotoxic T-cell activities although the mechanisms associated with this effect are not known.

HGFL shares a similar domain structure to hepatocyte growth factor (HGF). HGF is a fibroblast-derived growth factor that acts in a paracrine as well as autocrine manner to activate the c-Met receptor [31]. HGFL is primarily produced by hepatocytes and is secreted into the circulation, acting in an endocrine manner to stimulate Ron. While there is an abundance of studies regarding HGF activity, only a few reports have examined the importance of HGFL. Ectopic overexpression of HGFL in mammary tumor cells derived from polyoma middle T-antigen expressing mice promoted early tumor growth and broadened the spectrum of metastasis compared to control tumor cells [7]. Additionally, ectopic overexpression of HGFL increased metastasis of small cell lung carcinoma cells [32]. Although these studies suggest an important function for the overexpression of HGFL in tumor growth and metastasis, they fail to decipher the physiological relevance of endogenous levels of HGFL in tumorigenesis and metastasis. Prior studies in this area have shown that while HGFL deletion in normal mice does not affect circulating blood counts or differentials, examination of normal mammary gland development in HGFL−/− mice suggest that HGFL may play an important role as a chemoattractant for macrophages, with loss of HGFL associated with alterations in macrophage recruitment to the terminal end bud of the developing mammary gland [39].

Here, we show that MMTV-Ron mice lacking HGFL (MMTV-Ron^HGFL−/−^ mice) have a significant delay in the development of mammary hyperplasia and mammary tumor onset. This delay precedes a reduction in tumor size and reduced metastatic burden in the MMTV-Ron^HGFL−/−^ mice. Moreover, HGFL deficient tumors exhibited decreased cell proliferation and angiogenesis as well as increases in tumor cell death compared to HGFL proficient tumors. *In vitro* examination of epithelial cells derived from MMTV-Ron^HGFL−/−^ mice recapitulates the *in vivo* tumor cell intrinsic decreases in survival, migration and invasion compared to HGFL replete cells. Further, we show that cytotoxic T-cells are influenced by loss of HGFL, with T-cells from MMTV-Ron^HGFL−/−^ mice displaying increased proliferation and more efficient killing. In total, this study highlights the importance of HGFL in oncogenic Ron activation and mammary tumorigenesis through the regulation of both the primary tumor and the tumor microenvironment.

## RESULTS

### HGFL ablation delays the onset of mammary hyperplasia in MMTV-Ron mice

**Figure 1:**
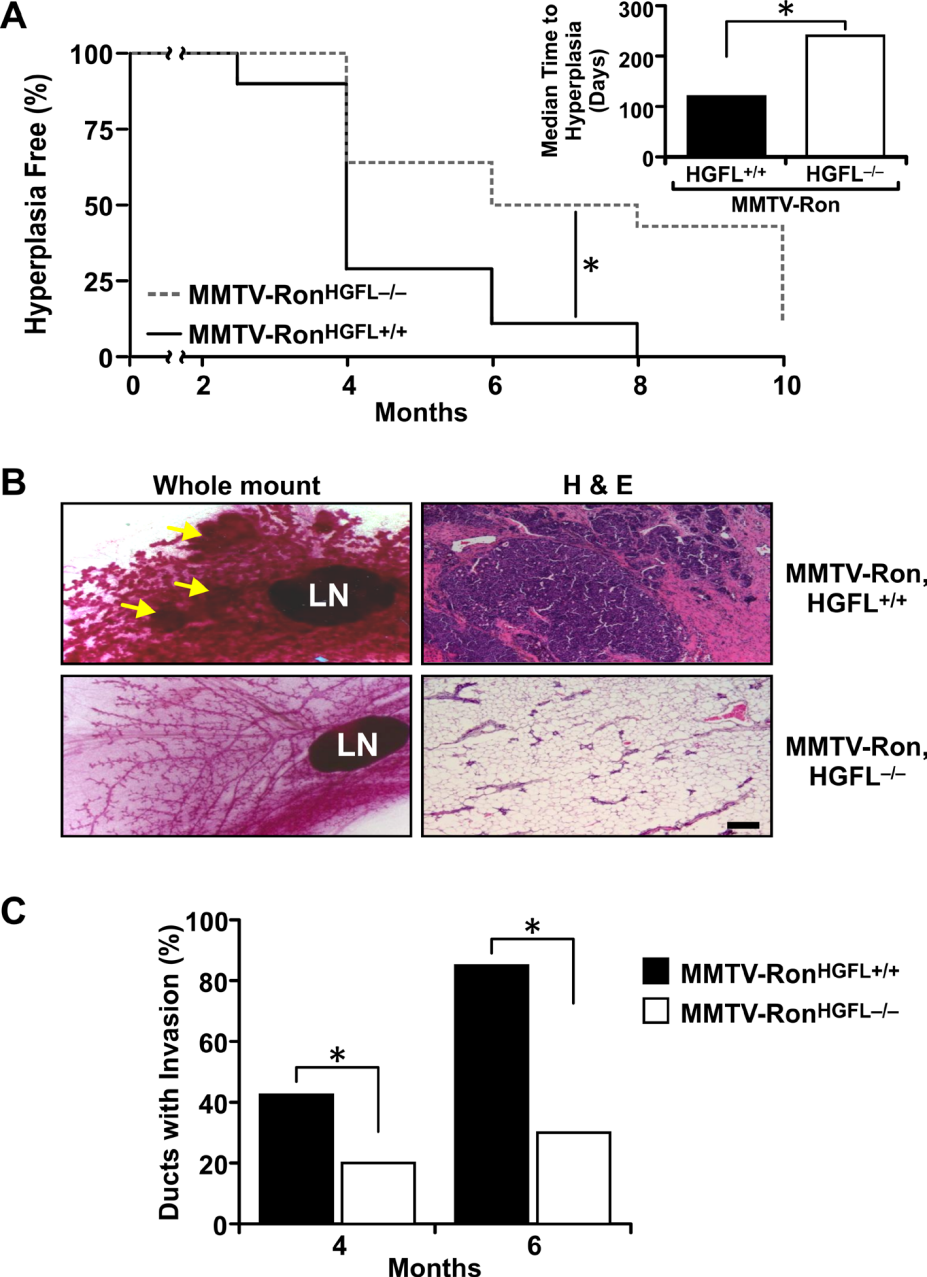
Mammary glands from MMTV-Ron^HGFL−/−^ mice exhibit delayed hyperplasia and invasive ductal carcinoma **A.** Quantification of mammary hyperplasia observed in inguinal mammary glands by whole mount and H&E analyses at 2.5, 4, 6, 8 and 10 months in MMTV-Ron^HGFL+/+^ and MMTV-Ron^HGFL−/−^ mice (*n* = 28–34/genotype). MMTV-Ron^HGFL−/−^ mice have a significant delay in the development of mammary hyperplasia, with a median time to hyperplasia at 240 days compared to 120 days in the MMTV-Ron^HGFL+/+^ mice, *P < 0.05. **B.** Representative mammary whole mounts and H&E sections from 8-month-old MMTV-Ron^HGFL+/+^ and MMTV-Ron^HGFL−/−^ mice. Scale Bar 100 μM. **C.** Quantification of invasive ductal carcinoma at 4 and 6 months. MMTV-Ron^HGFL−/−^ mice have significantly reduced local tumor invasion compared to MMTV-Ron^HGFL+/+^ mice (*n* = 20–30 sections per genotype per time point from 4–6 mice each). *P < 0.05.

To determine whether loss of HGFL changes kinetics of mammary tumorigenesis in MMTV-Ron mice, the MMTV-Ron^HGFL+/+^ mice were crossed to HGFL−/− mice. HGFL−/− mice are phenotypically normal with no differences observed in the number or morphology of circulating blood cells or platelets compared to control mice [33]. The contribution of HGFL on the incidence and development of hyperplasia was examined by isolating inguinal mammary glands at 2.5, 4, 6, 8 and 10 months from MMTV-Ron^HGFL+/+^ and MMTV-Ron^HGFL−/−^ mice. The presence of ductal hyperplasia was identified by mammary whole mount and histological analyses of glands from both genotypes (Figure 1). By 4 months of age, 75% of the MMTV-Ron^HGFL+/+^ mice exhibited ductal hyperplasia and by 8 months, all mammary glands from MMTV-Ron^HGFL+/+^ mice displayed hyperplasia. In contrast, by 8 months of age, only about 50% of the MMTV-Ron^HGFL−/−^ glands contained hyperplastic nodules and a significant difference in the median time to the development of hyperplasia was also noted between groups (Figure 1A). Representative whole mounts and histological sections at 8 months for each group are shown in Figure 1B. To further examine the role of HGFL in tumor progression, ductal invasion (defined as tumor cell invasion through the basement membrane) was quantified by determining the number of ducts with invasive carcinoma by histological analysis. Similar to the hyperplasia data, a significant decrease in local tumor cell invasion was apparent in the mammary glands of MMTV-Ron^HGFL−/−^ mice at 4 and 6 months compared to MMTV-Ron^HGFL+/+^ mice (Figure 1C).

### HGFL loss impedes mammary tumor formation and metastatic dissemination in MMTV-Ron mice

**Figure 2:**
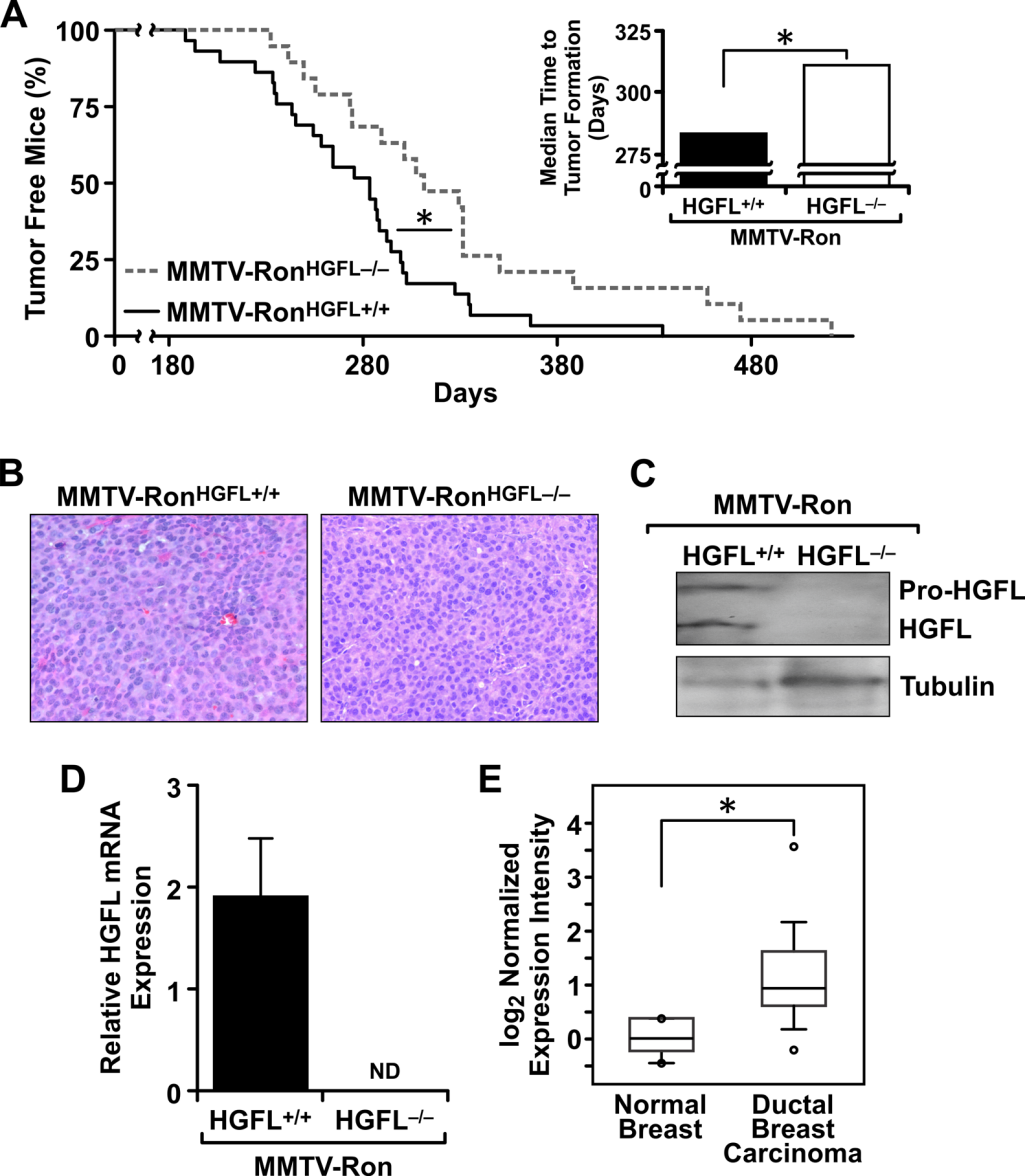
Mammary tumorigenesis is significantly delayed and metastasis reduced in MMTV-Ron^HGFL−/−^ mice **A.** MMTV-Ron^HGFL−/−^ mice (*n* = 19) have a significant delay in palpable tumor formation and the median time to palpable tumors (inset) compared to MMTV-Ron^HGFL+/+^ mice (*n* = 29). **B.** Representative histological images of mammary tumors from MMTV-Ron^HGFL+/+^ and MMTV-Ron^HGFL−/−^ mice are shown and were taken when tumors represented approximately 10% of body weight in each genotype. **C.** Mammary tumors from MMTV-Ron^HGFL+/+^ express HGFL protein while MMTV-Ron^HGFL−/−^ mice do not as depicted by Western analyses of tumor lysates. Tubulin is shown as a loading control. Both processed (active) and pro-HGFL are present in the mammary tumors of HGFL expressing mice. **D.** HGFL mRNA is expressed in MMTV-Ron^HGFL+/+^ mammary tumors while tumors from HGFL deficient animals do not express HGFL. (*n* = 5–6/glands from independent mice were examined/genotype). **E.** Oncomine data shows that HGFL expression is increased in ductal breast carcinoma compared to normal mammary gland. *P < 0.05. ND, not detectable.

The time to palpable tumor formation was followed in mice of each genotype. Although all mice developed mammary tumors, MMTV-Ron^HGFL−/−^ mice had a significant delay in the time to detection of palpable mammary tumors (Figure 2A). A significant increase in the median time to palpable tumor detection in MMTV-Ron^HGFL−/−^ mice was observed compared to MMTV-Ron^HGFL+/+^ mice (Figure 2A, inset). Tumor architecture, as examined by H&E staining, did not differ between groups (Figure 2B).

To examine HGFL loss, Western analysis was performed on mammary tumor lysates from both genotypes. HGFL protein was detected in tumors from MMTV-Ron^HGFL+/+^ mice (Figure 2C). Interestingly, HGFL mRNA expression was also observed in mammary tumors from MMTV-Ron^HGFL+/+^ mice suggesting that HGFL may be locally produced during mammary tumor progression (Figure 2D). To determine if HGFL expression is observed locally in human breast cancer, the Oncomine database was examined. Figure 2E depicts HGFL mRNA expression levels significantly upregulated in breast carcinomas compared to normal breast tissue [34]. Interestingly, examination of the cBioPortal datasets showed a significant co-occurrence of Ron and HGFL in breast cancer as well as in other cancer types. Further, in breast cancer, this database lists multiple mutations in HGFL that are associated with reduced survival (83.3 vs. 122.8 months in patients with and without a mutation respectively) [35, 36].

**Figure 3:**
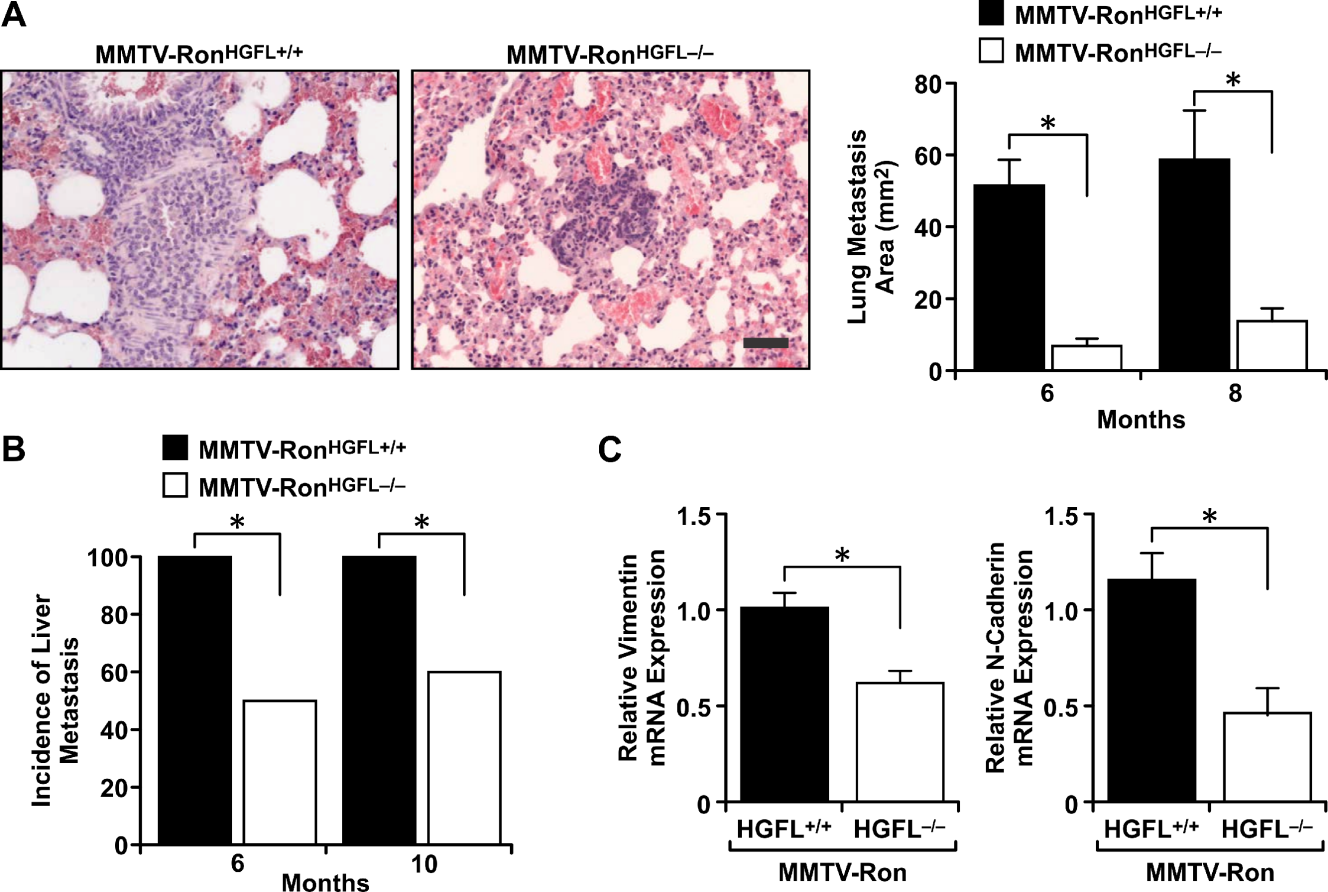
Lung and liver metastases are reduced in MMTV-Ron^HGFL−/−^ mice Lung and liver metastases were quantified by histological analysis at 6, 8, and 10 months of age. **A.** Lung tumor metastasis was significantly reduced in MMTV-Ron^HGFL−/−^ mice with the size of metastatic foci in MMTV-Ron^HGFL−/−^ mice significantly smaller compared to controls. The images show a representative metastatic lesion in the lungs of a MMTV-Ron^HGFL+/+^ mouse at 6 months while the image for MMTV-Ron^HGFL−/−^ is from 8 months (*n* = 5–10/group and time point) Scale bar = 100 μM. **B.** The incidence of liver metastases was significantly reduced in MMTV-Ron^HGFL−/−^ mice at 6 and 10 months of age (*n* = 5–10/group and time point). **C.** Mammary tumor tissue was examined by qRT-PCR for expression of EMT genes. N-cadherin and vimentin were significantly down regulated in MMTV-Ron^HGFL−/−^ mice (*n* = 3–4/grp). *P < 0.05.

MMTV-Ron^HGFL+/+^ mice exhibit a high metastatic burden [4] and a comparative analysis of metastasis was performed between genotypes. A significant reduction in the size of the metastatic foci in the lungs was found in the MMTV-Ron^HGFL−/−^ mice compared to MMTV-Ron^HGFL+/+^, with representative lung metastatic foci from the MMTV-Ron^HGFL+/+^ mice at 6 months compared to the smaller MMTV-Ron^HGFL−/−^ lung foci observed at 8 months (Figure 3A). In the liver, a significant reduction in the number of mice with liver metastasis was observed at all stages examined in HGFL deficient mice, with less than 60% of the MMTV-Ron^HGFL−/−^ livers displaying metastatic foci at 10 months (Figure 3B) compared to controls with 100% of mice exhibiting liver foci by 6 months of age. Accordingly, MMTV-Ron^HGFL−/−^ exhibit reduced expression of N-Cadherin and Vimentin, markers of epithelial to mesenchymal transition (EMT). (Figure 3C).

### HGFL deficient tumors have decreased proliferation, increased cell death and reduced angiogenesis

**Figure 4:**
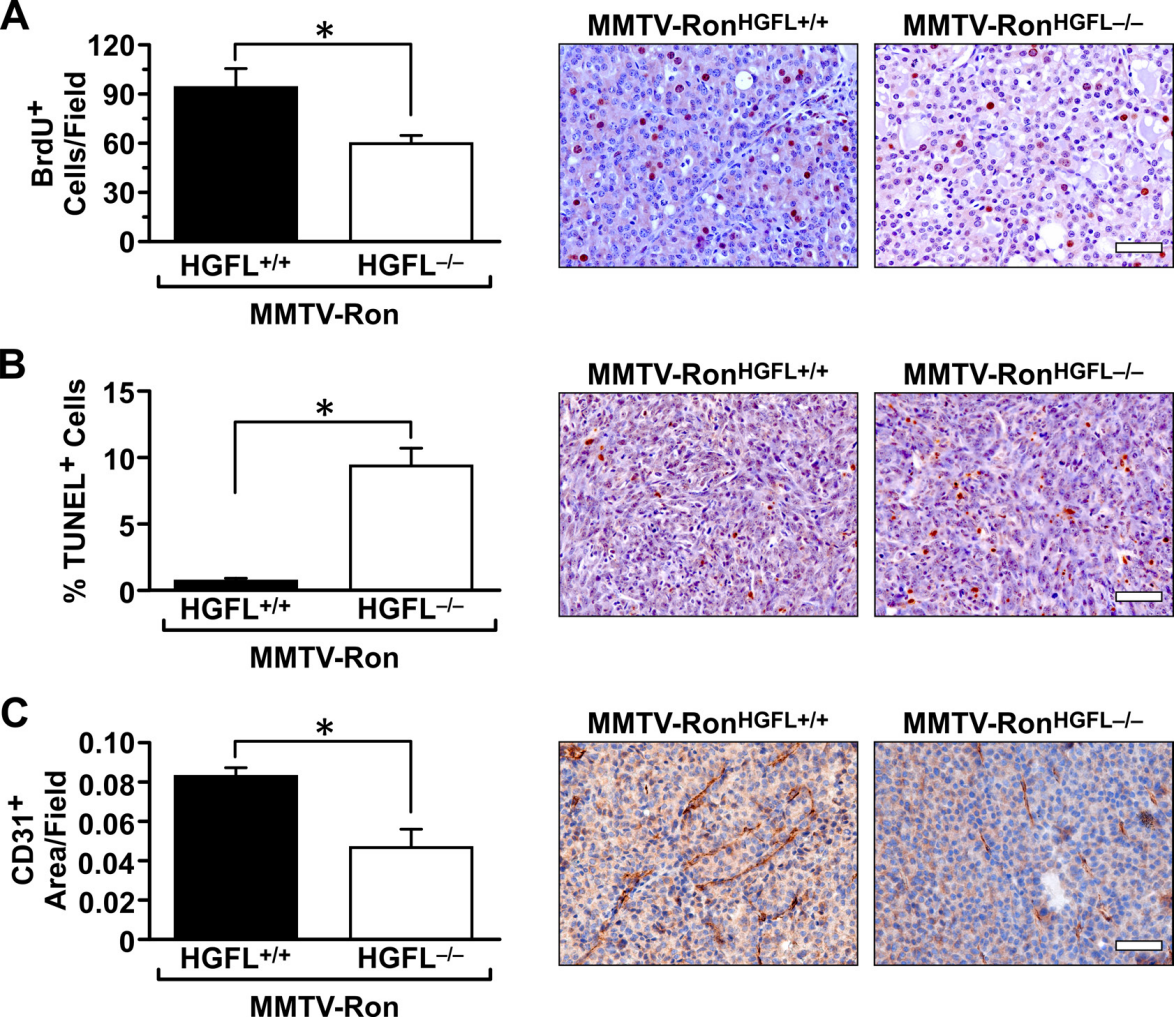
Mammary tumors from MMTV-Ron^HGFL−/−^ mice exhibit significantly reduced proliferation, increased cell death and reduced vessel staining Quantification of mammary tumor sections and representative images of BrdU, TUNEL and CD31stained sections. **A.** MMTV-Ron^HGFL−/−^ mice have significantly reduced number of proliferating tumor cells compared to MMTV-Ron^HGFL+/+^ tumors (*n* = 3 fields per mouse, 4 mice/group). **B.**, Mammary tumors from MMTV-Ron^HGFL−/−^ mice exhibit significantly more cell death than tumors from MMTV-Ron^HGFL+/+^ mice (*n* = 3 fields per mouse, 4 mice/group). **C.** Quantification of CD31 staining shows a significant reduction in vessel density in MMTV-Ron^HGFL−/−^ tumors compared to MMTV-Ron^HGFL+/+^ tumors. (*n* = 3 fields per mouse, 4 mice/group). *P < 0.05, Scale bar = 100 μM.

To examine the mechanism behind the delay in mammary tumorigenesis in the MMTV-Ron^HGFL−/−^ mice, mammary tumor sections, from mice when tumors were at similar sizes, were examined for the extent of mammary tumor cell turnover by BrdU and TUNEL staining. MMTV-Ron^HGFL+/+^ mammary tumors had significantly more proliferation based on BrdU incorporation than MMTV-Ron^HGFL−/−^ mice (Figure 4A). Mammary tumors from MMTV-Ron^HGFL−/−^ mice had a significant increase in TUNEL positive cells compared to tumors from MMTV-Ron^HGFL+/+^ mice (Figure 4B). Angiogenesis was also significantly blunted in the MMTV-Ron^HGFL−/−^ as indicated by CD31 staining of vessels (Figure 4C). These data suggest that loss of HGFL signaling influences tumor development through multiple mechanisms.

### Tumors from MMTV-Ron^HGFL−/−^ mice exhibit significant alterations in the tumor microenvironment

**Figure 5:**
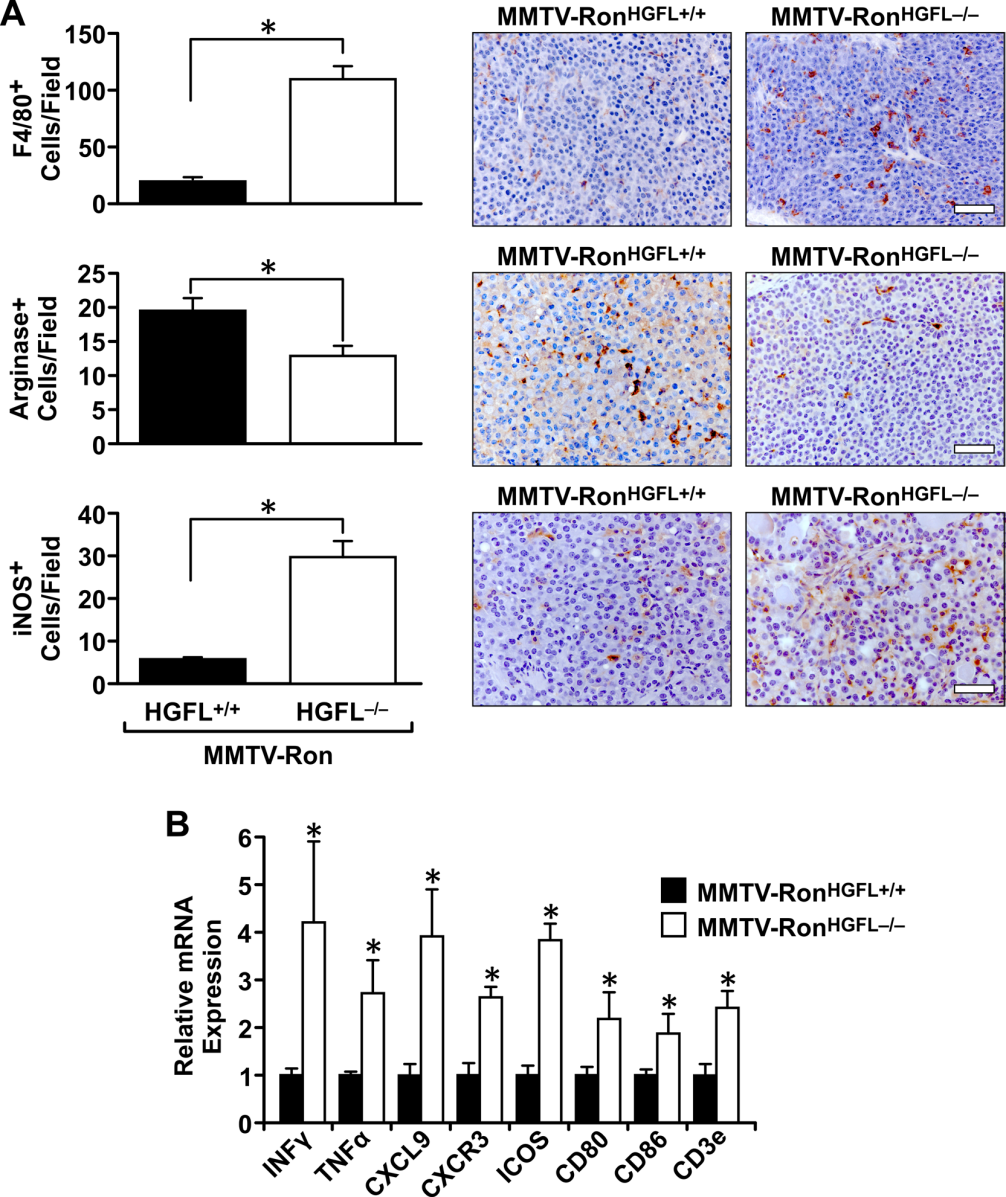
MMTV-Ron^HGFL−/−^ mice have an altered tumor microenvironment **A.** Quantification and representative images of F4/80, Arginase I and iNOS expression in MMTV-Ron mammary tumors by immunohistochemistry. Loss of HGFL increases macrophage infiltration and polarization toward a M1 phenotype, as shown by decreased Arginase I and increased iNOS staining. (*n* = 3 sections per mouse, 4 mice/group). **B.** Whole tumor tissue was examined by qRT-PCR for select cytokine and chemokine expression. An increase in inflammatory mediators within the tumor microenvironment of MMTV-Ron^HGFL−/−^ mice compared to control mice was apparent while the expression of T-cell co-stimulatory molecules are significantly increased. (*n* = 6 per group). *P < 0.05, Scale bar = 100 μM.

**Figure 6:**
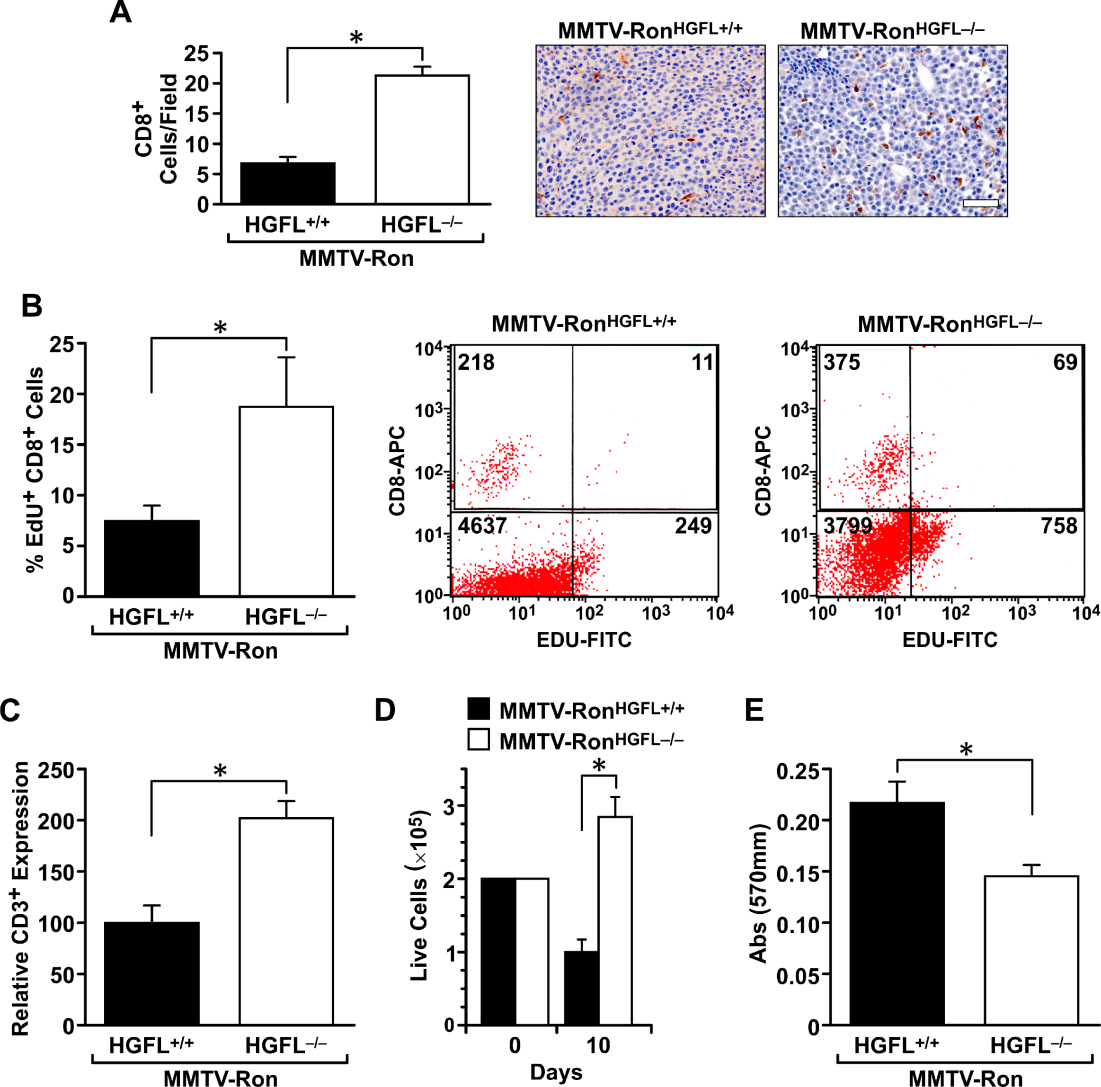
CD8+ T-cells are more abundant and display enhanced cytotoxic activities in MMTV-Ron^HGFL−/−^ mice **A.** Quantification and representative images of increased infiltration of CD8+ T-cells in MMTV-Ron^HGFL−/−^ tumors (*n* = 3 sections per mouse, 4 mice/group). **B.** Tumor infiltrating CD8+ T-cells are more proliferative as judged by EdU incorporation and flow cytometry. (*n* = 3–4 per group). A representative flow cytometry image of immune cells from mammary tumors of MMTV-Ron^HGFL+/+^ and MMTV-Ron^HGFL−/−^ mice examined for EdU and CD8+ T-expression. **C.** CD3 receptor expression, a marker of activated T-cells, is significantly increased in CD8+ T-cells from MMTV-Ron^HGFL−/−^ tumors versus MMTV-Ron^HGFL+/+^ tumors when measured by flow cytometry (*n* = 3–4 per group). **D.** Increases in splenic T-cell proliferation were observed *ex vivo* following CD3 stimulation for 10 days (*n* = 8 replicates per mouse, 2 mice per group). **E.** T-cells from MMTV-Ron^HGFL−/−^ versus MMTV-Ron^HGFL+/+^ mice have increased cytotoxic activities when co-cultured with mammary tumor cells from MMTV-Ron mice (R7 cells), as indicated by the reduction in cell number measured by crystal violet assays (*n* = 8 replicates per mouse, 2 mice per group). *P < 0.05, Scale bar = 100 μM.

As Ron is a known player in macrophage function, the distribution and activation status of tumor-associated macrophages was examined. A significant increase in F4/80 positive macrophages was measured in tumor sections from MMTV-Ron^HGFL−/−^ mice compared to controls (Figure 5A). This increase corresponded with a significant decrease in Arginase 1 staining (a M2 marker) and an increase in iNOS staining (a M1 marker), suggesting that loss of Ron signaling due to ligand deletion modifies tumor-associated macrophages toward a pro-inflammatory phenotype. These findings were further substantiated utilizing qRT-PCR, wherein mammary tumor samples from MMTV-Ron^HGFL−/−^ mice showed a significant increase in pro-inflammatory cytokine expression (Figure 5B). Along with the increase in M1 macrophage marker message expression, T-cell recruitment signals (CXCL9) along with co-stimulatory molecules (CD80/CD86) were increased in MMTV-Ron^HGFL−/−^ tumors (Figure 5B). These data suggest changes in macrophage polarization may drive changes in T-cell recruitment into the tumors of MMTV-Ron^HGFL−/−^ mice. This notion is supported by IHC data that shows a significant increase in CD8+ T-cells in tumors from HGFL deficient mice compared to controls (Figure 6A). To examine the proliferative capacity of the CD8+ T-cells, a subset of mice were injected with EdU 14 hours prior to euthanasia. Tumors were dissociated and isolated CD8+ T-cells were analyzed for EdU incorporation by flow cytometry. Figure 6B shows a significant increase in the percentage of EdU positive CD8+ T-cells in MMTV-Ron^HGFL−/−^ mice compared to HGFL expressing mice. Further, tumor-associated CD8+ T-cells from MMTV-Ron^HGFL−/−^ mice express more CD3e receptor, a marker of activation, than CD8+ T-cells from mammary tumors from MMTV-Ron^HGFL+/+^ mice (Figure 6C). To investigate changes in T-cell activity, splenocytes from tumor-bearing mice were utilized. Splenocytes isolated from tumor bearing MMTV-Ron^HGFL−/−^ mice exhibited significant increases in T-cell proliferation *ex vivo* (Figure 6D). Further, when cultured along with R7 tumor epithelial cells (derived from a mammary tumor from MMTV-Ron mice), splenic-derived T-cells from MMTV-Ron^HGFL−/−^ mice had increased cytotoxic activity compared to cells from MMTV-Ron^HGFL+/+^ mice as judged by a reduction in overall mammary tumor cell number (Figure 6E). Together, these data suggest that loss of HGFL leads to changes in macrophage polarization which alter the tumor microenvironment through increased recruitment, activation and cytotoxic function of CD8+ T-cells.

### Ron kinase activity is significantly reduced in MMTV-Ron^HGFL−/−^ mice, leading to diminished NF-κB and β-catenin signaling

**Figure 7:**
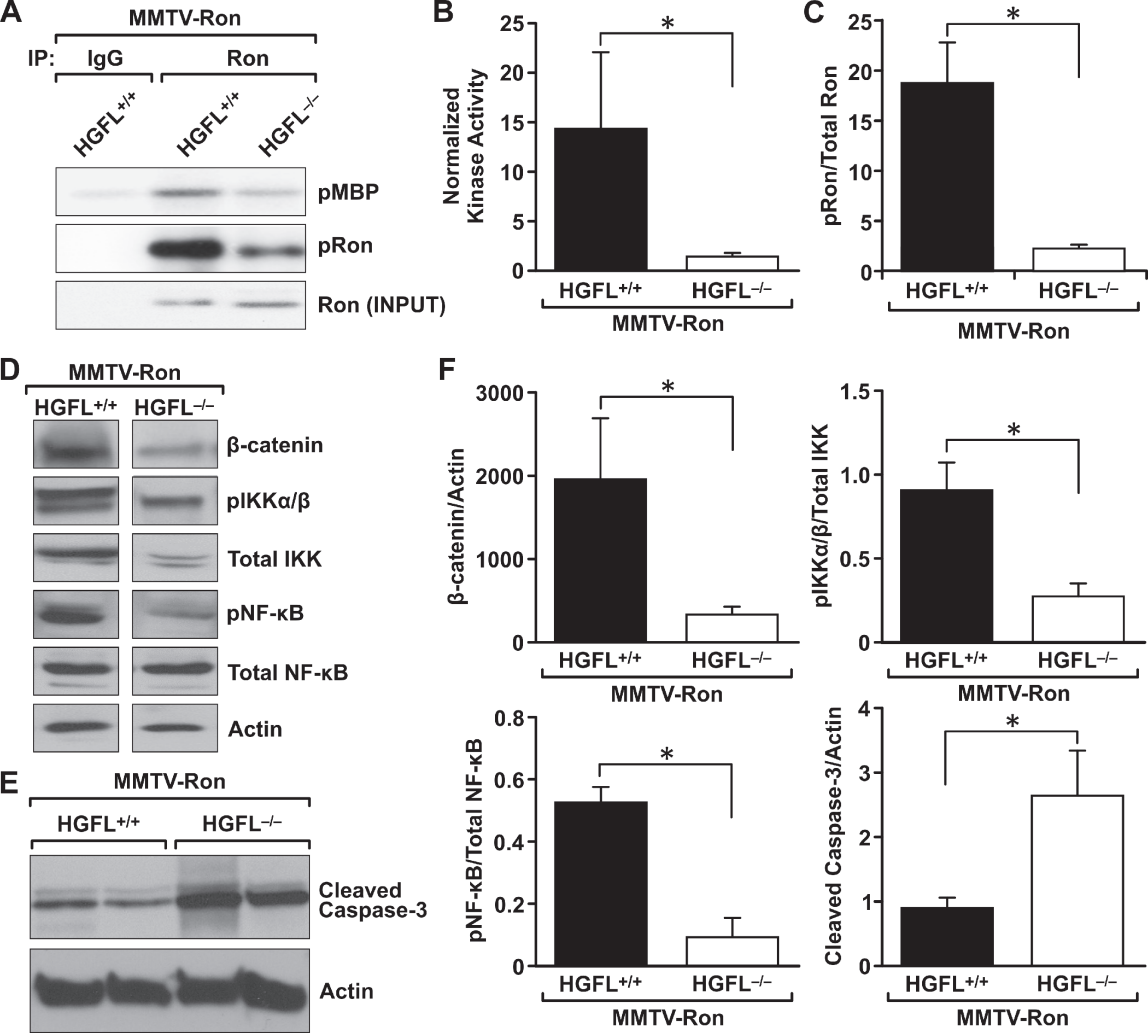
HGFL loss blunts Ron activation in mammary tumors from MMTV-Ron mice, leading to down regulation of β-catenin and the NF-κB pathway **A.** Ron activation status was determined by immunoprecipitating Ron from mammary tumor lysates from MMTV-Ron^HGFL+/+^ and MMTV-Ron^HGFL−/−^ mice and through kinase assays evaluating the ability of Ron to phosphorylate myelin basic protein (MBP). A representative image showing the extent of phosphorylated (p)MBP detected by autoradiography is shown. Western analysis of the amount of immunoprecipitated Ron (INPUT) and Ron phosphorylation (pRon) is shown. **B.** Graph shows quantification of Ron kinase activity from three independent kinase assays with *n* = 3 samples per group in each assay. The kinase activity is normalized to total immunoprecipitated Ron. **C.** Densitometric analysis of phosphorylated Ron over total Ron protein levels from mammary tumors from MMTV-Ron^HGFL+/+^ and MMTV-Ron^HGFL−/−^ mice. **D&E.** Western analysis of mammary tumor lysates from MMTV-Ron^HGFL+/+^ and MMTV-Ron^HGFL−/−^ mice for β-catenin and NF-κB pathway mediators D. and cleaved caspase 3 E. Actin serves as a loading control. **F.** Graphs show protein expression normalized to Actin or total protein by quantification of Western blot analyses (*n* = 4–6 mice per group). *P < 0.05.

To determine if activation of Ron is affected by HGFL loss, Ron kinase activity and phosphorylation status in mammary tumors from MMTV-Ron^HGFL+/+^ and MMTV-Ron^HGFL−/−^ mice was examined. Kinase assays were performed with equal amounts of Ron immunoprecipitated from mammary tumor lysates of MMTV-Ron^HGFL+/+^ and MMTV-Ron^HGFL−/−^ mice. Ron from MMTV-Ron^HGFL+/+^ tumors robustly phosphorylated an exogenous substrate (myelin basic protein, MBP) compared to Ron from MMTV-Ron^HGFL−/−^ tumors (Figure 7A–7B). Ron kinase activity was approximately 10-fold more active from HGFL replete mice compared to HGFL deficient mice. Further, a significant increase in the amount of phosphorylated Ron (Y1238/Y1239) receptor was found in MMTV-Ron^HGFL+/+^ mammary tumors as compared to MMTV-Ron^HGFL−/−^ tumors (Figure 7A, 7C).

Previous studies have demonstrated that β-catenin loss in MMTV-Ron mice delays the onset of mammary hyperplasia, time to palpable tumor formation and results in decreased metastasis [18, 37]. Additional studies have shown that *in vivo* tumor cell survival requires Ron-dependent NF-κB activation [17]. As the kinase activity and phosphorylation status of Ron were significantly reduced in the mammary tumors of the MMTV-Ron^HGFL−/−^ mice, the expression of downstream signaling pathways regulated in response to HGFL-mediated Ron activation was examined. Mammary tumor lysates from MMTV-Ron^HGFL+/+^ and MMTV-Ron^HGFL−/−^ mice were isolated and subjected to Western analysis. MMTV-Ron^HGFL−/−^ mammary tumors exhibited significantly reduced β-catenin levels compared to MMTV-Ron^HGFL+/+^ tumors. While no appreciable differences were observed in the activation of Akt, MAPK or STAT3 in the tumor lysates between genotypes (data not shown), several components of the NF-κB pathway were modified in MMTV-Ron^HGFL−/−^ tumors. Phosphorylated NF-κB was significantly decreased along with total and phosphorylated IKKα/β in the MMTV-Ron^HGFL−/−^ tumors compared to MMTV-Ron^HGFL+/+^ controls (Figure 7D). Cleaved caspase 3 was also increased in MMTV-Ron^HGFL−/−^ tumor lysates (Figure 7E), supporting the results of TUNEL staining in Figure 4B. Figure 7F depicts quantification of Western blot analyses. These data suggest that activation of the NF-κB pathway is dependent on HGFL-mediated Ron signaling and may drive changes in apoptosis.

### Tumor cell autonomous HGFL expression enhances the metastatic phenotype of Ron expressing breast cancer cells

**Figure 8:**
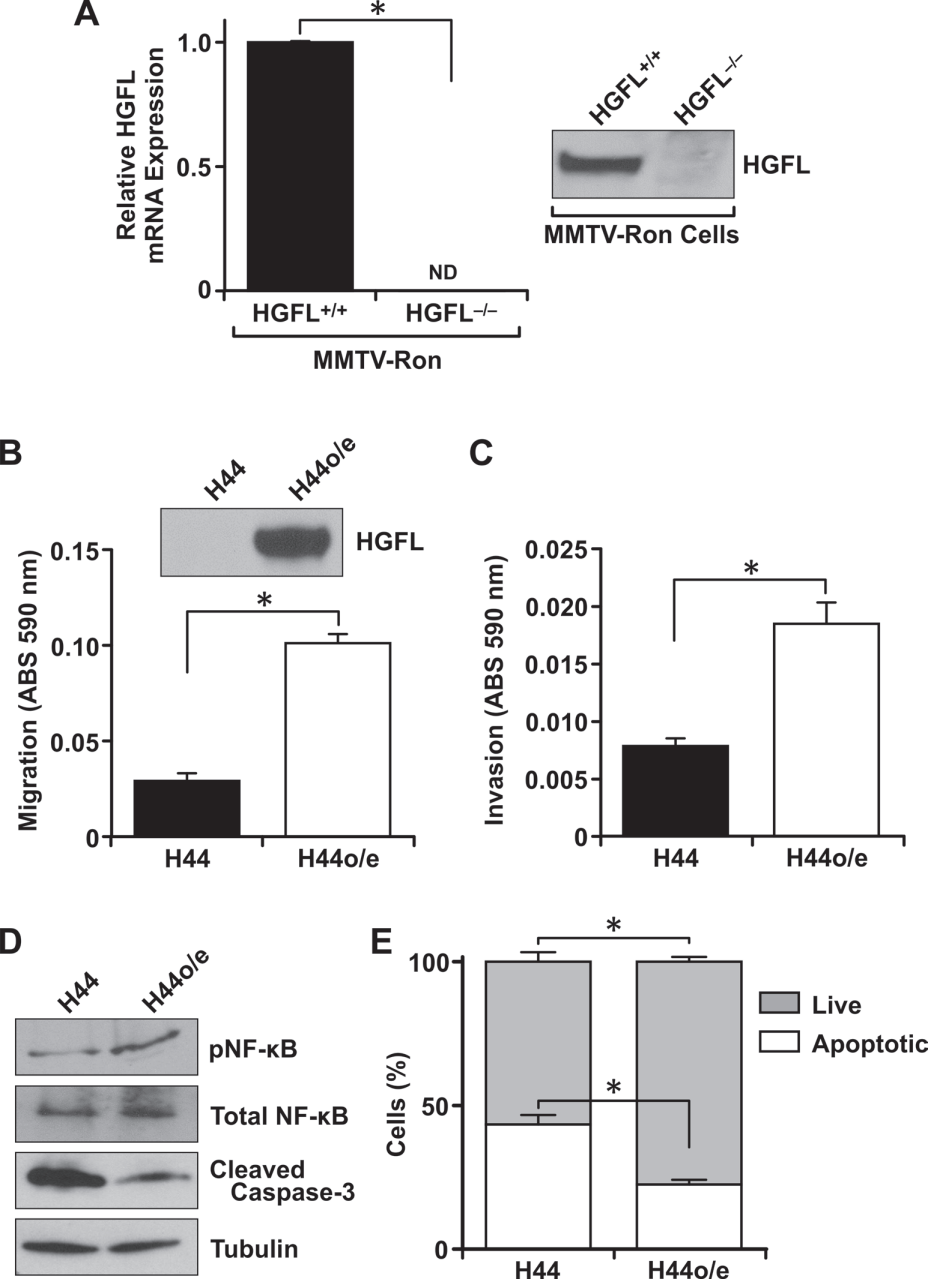
Tumor cell autonomous HGFL expression is important for mammary tumor cell migration, invasion and survival **A.** Expression of HGFL in mammary tumor cell lines derived from MMTV-Ron mice. Mammary epithelial cells from MMTV-Ron^HGFL+/+^ mice express and secrete processed HGFL while MMTV-Ron^HGFL−/−^ cells have no detectable expression of HGFL. **B–C.** H44 cells transduced to re-express HGFL (H44o/e) are significantly more migratory and invasive compared to HGFL deficient H44 cells (composite of *n* = 9 wells per group). **D.** Isolated epithelial cells with and without HGFL recapitulate the protein alterations observed from mammary tumors in MMTV-Ron mice with reduced NF-κB pathway activation observed in HGFL deficient cells. **E.** HGFL deficient cells (H44) are more apoptotic than those expressing HGFL by Annexin V/PI staining (composite of *n* = 9 wells per group). *P < 0.05, ND = not detectable.

As qRT-PCR data suggested that tumors from MMTV-Ron^HGFL+/+^ mice produce HGFL locally, mammary tumor epithelial cell lines were derived from MMTV-Ron^HGFL+/+^ and MMTV-Ron^HGFL−/−^ mice and further quantified for HGFL expression. Primary mammary tumor cells derived from MMTV-Ron^HGFL−/−^ mice (referred to as H44 cells) had no measurable HGFL message or protein expression (Figure 8A). However, significant HGFL expression was observed in primary mammary tumor cells from MMTV-Ron^HGFL+/+^ mice (Figure 8A). This is a novel discovery, as no reports have demonstrated tumor cell production and secretion of HGFL from breast cancer cells. To characterize the requirement for tumor cell derived HGFL in metastatic dissemination, H44 mammary tumor cells were transduced with control or HGFL producing lentiviral vectors to re-express HGFL. The HGFL overexpressing cells (H44o/e) produce and secrete HGFL (Figure 8B). While the growth of the H44 and H44o/e cells did not differ *in vitro* (data not shown), the H44o/e cells had significantly elevated migration (Figure 8B) and invasion (Figure 8C) compared to H44 cells. Further, in an examination of downstream signaling pathways, HGFL re-expression was able to promote NF-κB activation (Figure 8D) and β-catenin expression (data not shown). HGFL expression also augmented survival of the mammary tumor cells as judged by Annexin V-PI staining (Figure 8E). Consistent with promoting survival, HGFL re-expression also decreased cleaved caspase-3 protein (Figure 8D). Together, these data highlight the importance of tumor cell autonomous HGFL in the metastatic program.

## DISCUSSION

There are mounting reports implicating the Ron receptor tyrosine kinase in breast cancer progression and metastasis. However, there is scant information on whether Ron activation in this context requires its ligand, HGFL. Limited studies have highlighted the importance of HGFL in tumor models, including the mammary gland using ectopic overexpression [7, 32]. However, a major limitation of overexpression studies is that they do not investigate function within a physiological context. The study presented here is the first to examine the role of endogenous HGFL in the context of Ron overexpression, a condition associated with poor prognosis in human tumors [7]. In support of this data, mammary gland specific overexpression of HGF (the ligand for c-Met) in transgenic mice induces mammary hyperplasia that progressed to form invasive mammary tumors compared to wild type mice [38]. Akin with overexpression studies, our data demonstrates the importance of HGFL in promoting ductal hyperplasia and progression to tumorigenesis in MMTV-Ron mice. Additionally, HGF overexpressing mammary tumors led to increased phosphorylation of the c-Met receptor [38]. Similarly, decreased phosphorylation and activity of the Ron receptor was observed in MMTV-Ron^HGFL−/−^ tumors compared to MMTV-Ron^HGFL+/+^ tumors. These data highlight the importance of endogenous HGFL in Ron activation during mammary tumorigenesis.

Previous studies have documented that Ron or HGFL overexpression is associated with heightened metastasis [4–7, 15]. In this report, both local invasion as well as metastatic dissemination was examined temporally. Interestingly, evidence of tumor cells invading into the surrounding mammary gland (invasive ductal carcinoma) were identified in both MMTV-Ron^HGFL+/+^ and MMTV-Ron^HGFL−/−^ glands as early as 4 months. However, MMTV-Ron^HGFL+/+^ mice had a significantly higher percentage of ducts exhibiting local invasion at all measured time points compared to MMTV-Ron^HGFL−/−^ animals. Consistent with the high invasive capacity of Ron expressing mammary tumors, 100% of mice examined in the MMTV-Ron^HGFL+/+^ group had metastasis to the lungs and liver at 8 and 10 months. In comparison, significantly fewer MMTV-Ron^HGFL−/−^ mice had liver metastasis at these time points (50% at 6 months and 60% at 10 months). While the incidence of lung metastases was similar between groups, metastatic foci were significantly smaller in the MMTV-Ron^HGFL−/−^ lungs compared to lungs from MMTV-Ron^HGFL+/+^ mice. These results are similar to that reported in the polyoma middle T-antigen expressing mouse mammary tumor model, where ectopic overexpression of HGFL led to an increased metastatic burden compared to control mice [7]. Further studies have shown that Huh7 liver cells treated with exogenous HGFL display epithelial-mesenchymal transition (EMT) markers *in vitro* [40], consistent with the data herein demonstrating reduced EMT marker expression in HGFL deficient tumors compared to HGFL replete tumors. Together, these data highlight the importance of endogenous HGFL in the activation of Ron in cancer cell invasion and metastasis.

An interesting finding of these studies was the alterations observed in the tumor microenvironment. In the current study, loss of HGFL regulated the recruitment and polarization of tumor-associated macrophages, similar to recent studies using Ron signaling deficient mice showing decreased tumor burden associated with increased M1 macrophage infiltration and cytotoxic T cell activity [16, 30]. With loss of Ron signaling, the tumor-associated macrophages expressed markers of classically activated (M1) macrophages and were associated with significant increases in proinflammatory cytokines, as well as markers of cytotoxic T-cells. T-cell numbers were significantly increased in MMTV-Ron^HGFL−/−^ mice, with tumor-associated CD8+ T-cells proliferating more than those from MMTV-Ron^HGFL+/+^ mice. Further investigation showed increased cytotoxic activity of T-cells in the MMTV-Ron^HGFL−/−^ mice. Increases in tumor-infiltrating lymphocytes, including CD8+ T-cells, are widely associated with improved outcomes in human breast cancer patients [41–45].

In addition to immune cells, HGFL loss also had a profound effect on the formation of vessels within the tumor microenvironment. These results are similar to published studies that describe Ron expression in the tumor proper as a promoter of tumor angiogenesis. *In vitro*, knockdown of Ron expression in prostate cancer cells reduced the production of angiogenic chemokines (CXCL1, 5 and 8) leading to a decrease in endothelial cell migration [19]. Correspondingly, *in vivo* implantation of Ron knockdown cells in mouse xenograft experiments lead to decreased tumor growth and angiogenesis compared to Ron expressing cells. Further, conditioned media from Ron expressing pancreatic cells induced microtubule formation in human microvascular endothelial cells [46]. Together these data show that activation of Ron signaling within tumor cells plays an important role in mediating vessel formation in the growing tumor. Similarly, our data show a decrease in CD31 staining in MMTV-Ron^HGFL−/−^ mice, indicative of reduced vessel density compared to control mice.

The data herein are the first to suggest that HGFL is playing an important role in cross talk between multiple cell types within the tumor microenvironment. As the Ron receptor is a target of broad-spectrum kinase inhibitors and specific antibodies currently in clinical trials, our data provide strong pre-clinical support for targeting this signaling pathway and more specifically for targeting HGFL in breast cancer. Further, targeting of HGFL-Ron signaling may provide the signals needed to reactivate the anti-tumor immune response, leading to an effective therapy for breast cancer and other Ron expressing tumor types.

Many pathways are activated by the Ron receptor tyrosine kinase, including β-catenin PI3K/Akt, MAPK, NF-κB and STAT3. In the context of this study, loss of HGFL under conditions of Ron overexpression led to alterations in the NF-κB pathway and a reduction in β-catenin. β-catenin is a component of the Wnt signaling pathway and is critical for multiple cell functions. HGFL dependent Ron signaling has been shown to stimulate β-catenin nuclear localization and transcriptional activity in human and mouse breast cancer cell lines *in vitro* and *in vivo* [18, 37, 47]. The results described herein extend these observations and further strengthen the connection between Ron signaling and β-catenin, whose deregulated expression is associated with poor prognosis in breast cancer. These data are consistent with published studies showing β-catenin nuclear accumulation in WAP-HGF mammary tumors [38], suggesting a strong association between HGFL expression and β-catenin signaling. The NF-κB signaling pathway is known to activate a vast number of target genes, generally thought as pro-survival factors which support cancer development [48]. Knockdown of Ron signaling using siRNA in prostate tumor cell lines showed increased accumulation of the NF-κB inhibitory protein IκBα as well as reduced NF-κB activity [19]. These changes were associated with a reduction in angiogenic chemokines and reduced vessel density. Our data show a reduction of the NF-κB pathway through reduced NF-κB and IKKα/β phosphorylation as well as reduced CD31 staining. The reduction of NF-κB signaling suggests reduced activation of downstream target genes. Some of these target genes include those involved in apoptosis, such as X-link inhibitor of apoptosis protein (XIAP), an inhibitor of caspase-3 and 7 [49]. An increase in cleaved caspase-3 protein was observed in mammary tumors of MMTV-Ron^HGFL−/−^ mice compared to MMTV-Ron^HGFL+/+^ mice, along with increased apoptosis as measured by TUNEL assays. These data are supported by *in vitro* HGFL re-expression assays in HGFL deficient cells, where HGFL expression lessened cleaved caspase-3 protein expression and apoptosis measured by Annexin V/PI flow cytometry. In the context of mammary tumorigenesis, our results suggest that HGFL may promote mammary tumorigenesis and metastasis through a mechanism associated with increased tumor cell survival mediated by NF-κB and β-catenin signaling.

Importantly, this is the first study to describe the tumor cell autonomous production of HGFL from mammary tumors cells. Recent publications from our group have shown that HGFL expression is present in the developing mammary gland as well as in prostate tumor cells [39, 50]. In this study, we demonstrate heightened HGFL mRNA expression during tumorigenesis. The source of local HGFL production is the tumor epithelial cells, which was confirmed in cell lines derived from Ron overexpressing tumors. As the liver maintains circulating HGFL at a high concentration, the production of locally derived HGFL suggests that local HGFL may play a crucial role in tumorigenesis by influencing multiple cell types within the tumor microenvironment. Functionally, studies in this report demonstrate that tumor cell autonomous HGFL production, in isolated mammary tumor epithelial cells, is sufficient for enhanced invasion, migration and survival *in vitro*. These *in vitro* studies clearly support the *in vivo* findings of decreased metastatic capability of mammary tumor cells lacking HGFL as well as reduced EMT gene expression in mammary tumors of MMTV-Ron^HGFL−/−^ mice, suggesting that tumor cell produced HGFL is of critical importance within the developing tumor. However, the extent to which additional tumor models and human tumors exhibit HGFL upregulation and invasive growth remains to be determined.

The regulation of Ron activation and subsequent signaling by HGFL represent a novel and specific target for cancer therapeutics. The only known receptor for HGFL is Ron and despite sequence homology, HGF and HGFL are not cross-reactive [9]. As such, HGFL is an attractive therapeutic candidate for targeting the Ron receptor pathway. Several broad-spectrum receptor tyrosine kinase inhibitors with some affinity for Ron are already in phase I-II clinical trials. These drugs have shown effectiveness in preclinical models [51, 52] and Foretinib has been shown to reduce phosphorylation of Ron in human patient samples [52]. The data presented here clearly implicate the importance of HGFL for activation of Ron signaling in tumor formation and metastatic dissemination, suggesting the targeting of Ron, either through current receptor tyrosine kinase inhibitors or through blockade of HGFL binding may be suitable for inhibiting Ron activated downstream signaling. Further, inhibition of Ron signaling drives macrophage polarization toward a pro-inflammatory phenotype, which may allow for immune cell recognition of tumor cells and facilitate immune mediated tumor killing through both macrophages and cytotoxic T-cells.

In conclusion, we show that HGFL expression enhances Ron-driven mammary tumorigenesis. Loss of HGFL delays mammary tumor formation and progression along with metastatic dissemination. These delays are associated with reduced Ron phosphorylation and Ron activity that manifest as downregulation in β-catenin and NF-κB signaling. Further, the reduction in tumor burden is associated with increased apoptosis in tumors as well as isolated mammary epithelial cells lacking HGFL. We describe changes within immune cells of the tumor microenvironment, whose increased activity may be causal in the diminution of tumor burden seen in the MMTV-Ron^HGFL−/−^ mice. Finally, we demonstrate that tumor epithelial cells are capable of producing and secreting HGFL, suggesting that tumor cell produced HGFL is playing a major role in Ron activation and subsequent tumorigenesis. The data presented here provide the rationale for the development of targeted therapies that inhibit HGFL binding and subsequent activation of Ron.

## MATERIALS AND METHODS

### Generation of mice

To generate MMTV-Ron^HGFL−/−^, FVB female HGFL−/− mice (backcrossed 9 generations to FVB) [33] were crossed to FVB MMTV-Ron positive males. The resultant heterozygous males (MMTV-Ron+^HGFL+/−^) were backcrossed to HGFL+/− females to generate the females used in this study. Female mice were maintained as described previously [4]. Genotyping of transgenic mice was performed by PCR analysis. Primers sets for identification of MMTV-Ron and HGFL−/− mice are as follows: MMTV-Ron forward 5′-TGG GTG GTG AGG TCT GCC AAC ATG A-3′, reverse 5′-CCG TCT TCG GGA GTT AAA GAT CAG GG-3′. For HGFL, the following 3 primers were utilized: 5′-AAT CTG GGT TGC CAG TTA ACT TTG TGT-3′, 5′-AAG TTC TCT TCC AGG CCA TTC TTT GGC-3′, 5′-GGA AAA GCG CCT CCC CTA CCC GG-3′.

### Mammary tumor initiation and progression

Females were palpated weekly to assess mammary tumor development as previously described [4]. At the designated time points, frozen and formalin fixed tissues were collected from thoracic and inguinal mammary glands, lungs and liver. All animal procedures were approved by the University of Cincinnati Institutional Animal Care and Use Committee.

### Cell culture

Mammary epithelial tumor cell lines from MMTV-Ron^HGFL+/+^ mice (R7 cells) and MMTV-Ron^HGFL−/−^ mice (H44 cells) were derived as previously described [4, 53]. H44 cells were infected with lentiviral vector constructs designed to re-express HGFL (H44o/e). HGFL secretion from stably transduced cells was verified using western analysis (described below).

### *In vitro* assays

All assays were performed in triplicate and repeated at least 3 times. Boyden chambers were used for migration and invasion assays, with the addition of 1:1 matrigel: media (BD Biosciences, San Jose, CA) coating the top well for invasion assays. MTT (3-(4, 5-Dimethylthiazol-2-yl)-2, 5-diphenyltetrazolium bromide, Sigma Aldrich, St, Louis, MO) was used to quantify cell viability at 0, 24 and 48 hours. Annexin V and propidium iodide (BD Biosciences) staining was used to measure cell death on serum-starved cells at 24 hours.

### Tissue histology

Tissues were processed as described previously [4]. Briefly, mammary glands, lungs, and liver samples were fixed, paraffin embedded, and cut into in 4 μm sections. Sections were stained with hematoxylin and eosin for routine histological examination. Whole mount analysis was completed as previously described [4].

For BrdU analysis, mice were injected with 5-bromo-2′-deoxyuridine (BrdU) 2 hours prior to euthanasia and immunohistochemistry was performed as previously described [54]. In Situ Apoptosis Detection Kit (EMD Millipore, Billerica, MA) was used according to the manufacturer's instructions for TUNEL staining. For both proliferation and apoptosis, quantification of positive epithelial cells at 400X magnification was completed by counting at least three independent fields per slide from at least three different tumors from each group.

Standard procedures were used for staining of F4/80 (eBiosciences, San Diego, CA), Arginase I (BD), iNOS (BD Biosciences) and CD8a (BD Biosciences), with the number of positive cells counted in at least three independent fields per slide from at least three different tumors per group. CD31 (Dako, Carpinteria, CA) was stained using standard procedures and quantified using image J software to calculate area stained in at least three independent fields per slide from at least three different tumors from each group.

### Kinase assays, western blotting and quantitative real time (qRT)-PCR

Kinase assays were performed according to published protocols [4]. In brief, Ron was immunoprecipitated from 500 μg mammary tumor lysates from MMTV-Ron^HGFL+/+^ and MMTV-Ron^HGFL−/−^ mice with 2 μg of primary antibody (anti-Ron C-20, Santa Cruz Biotechnology, Santa Cruz, CA, USA). Equal amounts of immunoprecipitated Ron were placed in a kinase reaction with equal concentrations of myelin basic protein (MBP) (Millipore, Billerica, MA, USA) as substrate and [γ–32P]-ATP as the phosphate donor. Following incubation, samples were separated using sodium dodecyl sulfate–polyacrylamide gel electrophoresis and the gels were fixed, dried and imaged on a phosphoimager to detect labeled MBP. Kinase activity was normalized to total immunoprecipitated Ron following Western analysis (INPUT) of immunoprecipitated Ron. For measurement of secreted HGFL, cells were seeded at an equal density and incubated for 36 hours in serum free media. The conditioned media from these cells was collected and concentrated using Amicon Ultracel filter units with 3kD membranes (EMD Millipore). For total tissue lysates, the following antibodies were used: phospho-NF-κB, phospho-IKKα/β, total NF-κB, total IKK, cleaved caspase-3, phospho-Stat3, and β-catenin (Cell Signaling Technology, Danvers, MA) as well as HGFL (Santa Cruz Biotechnology, Dallas, TX). β-Actin (Cell Signaling) or α-tubulin (Santa Cruz) expression was measured as a loading control. qRT-PCR was performed as previously described [25] and primer sets used are as follows:

18S (AGTCCCTGCCCTTTGTACACA, GATCC GAGGG CCTCACTAAAC);
CD80 (ACCCCCAACATAA CTGAGTCT, TTCCAACC AAGAGAAGCGAGG);
CD86 (CTTACGGAAGCACCCACGAT, TCTCCACGG AAAC AGCATCT);
CXCL9 (GGAGTTCGAGGAACCCTAGTG, GGGATTT GTAGTGGATCGTGC);
CXCR3 (TACCTTGAGGTTAGTGAACGTCA, CGCTC TCGTTTTCCCC ATAATC);
HGFL (GCTGTGGCATCAAAACCT, TGGAAAGGGTG CGAGT);
iCOS (TGACCCACCTCCTTTTCAAG, TTAGGGTCAT GCACACTGGA);
TNFα (GGTCCCC AAAGGGATGAGAA, CTCCAGCT GCTCCTCCACTT);
Vimentin (CCAACCTTTTCTTCCCTGAA, TGAGTG GGTGTCAACCAGAG);
N-Cadherin (AGCGCAGTCTTACCGAAGG, TCGCTG CTTTCATACTGAACTTT)
IFNγ (GATATCTCGAGGAACTGGCAAAA, CTTCAAA GAGTCTGAGGTAGAAAGAGATAAT).

### T-cell isolation and EdU analysis

For EdU analysis of tumor associated CD8+ T-cells, mice were injected with 5-ethynyl-2′-deoxyuridine (EdU) 14 hours prior to euthanasia. Single cell suspensions of mammary tumors were obtained using enzymatic and mechanical dissociation followed by immune cell fraction isolation using gradient centrifugation. Immune cell fractions were sorted by flow cytometry for the isolation of purified CD8a+ T-cells (CD8a-APC, eBiosciences). Sorted CD8+ T-cells were stained for CD3-PE (eBiosciences) and measured using flow cytometry. Co-staining for EdU incorporation (FITC) and CD8a expression was completed according to manufacturer's instructions (Life Technologies, Grand Island, NY) and analyzed by flow cytometry. Splenocytes were isolated using mechanical separation of whole spleens followed by elimination of red blood cells using erythrocyte lysis buffer. For proliferation, cells were plated on CD3e antibody coated plates (BD Biosciences) and counted using both manual counting and OD values from crystal violet stained cells. For cytotoxicity assays, splenocyte derived T-cells were co-cultured with R7 mammary tumor cells. After 24 hours, cells were washed to remove T-cells and dead tumor cells, the remaining viable cells were fixed in 4% paraformaldehyde. Following fixation, cells were stained with crystal violet and cell viability read at Abs (570 mm).

### Statistical analysis

Statistical analysis for hyperplasia was determined using *z*-test. Data on tumor development was subjected to Kaplan-Meier analysis using a log-rank test. Incidence data (invasive ductal carcinoma and metastasis incidence) were analyzed using Fisher's Exact Test. All other data are represented as the mean ± SE and were analyzed with Student's *t*-tests using GraphPad Prism software (San Diego, CA). Significance was set at *P* < 0.05.
